# The Role of Purine Metabolites as DAMPs in Acute Graft-versus-Host Disease

**DOI:** 10.3389/fimmu.2016.00439

**Published:** 2016-10-21

**Authors:** Petya Apostolova, Robert Zeiser

**Affiliations:** ^1^Department of Hematology, Oncology and Stem Cell Transplantation, University Medical Center, Albert Ludwig University of Freiburg, Freiburg, Germany

**Keywords:** graft-versus-host disease, P2X7, P2Y2, ATP, ectonucleotidase, inflammasome, uric acid, neutrophils

## Abstract

Acute graft-versus-host disease (GvHD) causes high mortality in patients undergoing allogeneic hematopoietic cell transplantation. An early event in the classical pathogenesis of acute GvHD is tissue damage caused by the conditioning treatment or infection that consecutively leads to translocation of bacterial products [pathogen-associated molecular patterns (PAMPs)] into blood or lymphoid tissue, as well as danger-associated molecular patterns (DAMPs), mostly intracellular components that act as pro-inflammatory agents, once they are released into the extracellular space. A subtype of DAMPs is nucleotides, such as adenosine triphosphate released from dying cells that can activate the innate and adaptive immune system by binding to purinergic receptors. Binding to certain purinergic receptors leads to a pro-inflammatory microenvironment and promotes allogeneic T cell priming. After priming, T cells migrate to the acute GvHD target organs, mainly skin, liver, and the gastrointestinal tract and induce cell damage that further amplifies the release of intracellular components. This review summarizes the role of different purinergic receptors in particular P2X7 and P2Y2 as well as nucleotides in the pathogenesis of GvHD.

## Introduction

Allogeneic hematopoietic cell transplantation (allo-HCT) is a potentially curative therapeutic option mainly for patients with acute leukemias or malignant lymphomas but also for selected non-malignant diseases. Initial transplantation attempts remained ineffective due to the lack of knowledge regarding human leukocyte antigen (HLA) compatibility between donor and recipient and the lack of adequate immunosuppressive drugs. Today, more than 60 years later, immunologic reactions between donor and host still remain one of the major causes of morbidity and mortality after allo-HCT. Here, we discuss the impact of purines as danger-associated molecular patterns (DAMPs) in the context of acute GvHD. We decided to focus on purines and their receptors in GvHD because other danger signals in the context of allo-HCT have been discussed in a previous review (1). Nucleosides and nucleotides bind to the P1 and P2 family of purinergic receptors. Whereas adenosine activates four receptors belonging to the P1 family, UDP (Uridine-5′-diphosphate), uridine-5′-triphosphate (UTP), adenosine diphosphate (ADP), and adenosine triphosphate (ATP) activate the large family of P2 receptors with a variable affinity. The P2 receptor family is divided into two subfamilies, the ligand-gated ion channels P2X receptors and the G-protein-coupled P2Y receptors. Purinergic signaling is regulated by the expression of cell surface enzymes known as ectonucleotidases, most prominently CD39 and CD73 that convert ATP/UTP to ADP/UDP and ultimately to the respective nucleosides adenosine and uridine. Purinergic signaling modulates inflammation on multiple levels and contributes to the pathogenesis of a broad variety of diseases besides GvHD. P1 and P2 receptors show a variable distribution among different tissues that ensures a broad spectrum of effects. For instance, the P2Y2 receptor is expressed on immune cells but also on epithelial and endothelial cells and osteoblasts. Expression of the P2X7 receptor is predominant on immune cells, such as antigen-presenting cells, but is also found in the skin and pancreas (2). Expression of the P2Y12 receptor on platelets is a key feature for the use of P2Y12 receptor antagonists in the clinic.

## Purinergic Signaling in Cardiovascular Disease

Purinergic signaling has a well-established role in cardiovascular disease on multiple levels. For instance, nucleotides play a role in the formation of atherosclerotic plaques as a result of lipid metabolism dysregulation. In a murine model of atherosclerosis with apolipoprotein E-deficient mice, lack of the P2Y12 receptor was linked to a reduced plaque lesion area, decreased monocyte infiltration, and enhanced fibrous content of the plaque (3). In this same model, deficiency of the P2Y1 receptor significantly decreased the expression of vascular adhesion molecules P-selectin, VCAM-1, and ICAM-1 leading to diminished recruitment of leukocytes to lesion sites (4). Furthermore, endothelial cell cytoskeleton, motility, and adhesion are regulated via activation of the P2Y2 receptor following ATP or UTP binding (5). This is of particular importance due to the fact that the P2Y2 receptor is upregulated in the neointima of injured arteries in rats (6). Nucleotide binding to the P2Y2 receptor results in co-localization of the P2Y2 and VEGFR2 with subsequent upregulation of VCAM-1 that facilitates leukocyte adhesion (7). Endothelial cell migration is enhanced upon binding of ADP to the P2Y1 receptor via activation of the mitogen-activated protein kinase pathways (8). Last but not least, purinergic signaling has long been known to modulate platelet aggregation (9), mostly by ADP binding to the P2Y12 receptor (10). This fact led to the utilization of P2Y12 receptor antagonists, such as clopidogrel for inhibition of platelet aggregation for multiple cardiovascular diseases in patients (Table 1). Taken together, these data indicate that release of nucleotides with subsequent activation of purinergic receptor is a pro-inflammatory stimulus that enhanced leukocyte binding to the endothelium, platelet aggregation, and subsequent atherosclerotic plaque formation. Given the fact that multiple receptors have been implied to play a role, a more general purinergic receptor blockade might be required in order to achieve optimal protection.

**Table 1:** **Purinergic signaling and ectonucleotidases in inflammatory diseases**.

Disease context	Mechanism	Reference
Airway inflammation	ATP triggers airway inflammation via P2X7 expression on dendritic cells	(11, 12)
Airway inflammation	P2Y6 receptor expressed on lung epithelial cells mediates IL-6 and IL-8 secretion upon allergen challenge	(13)
Airway inflammation	ATP activation of the P2Y2 receptor contributes to eosinophilic lung inflammation	(44, 45)
Cardiovascular disease	P2Y12 receptor deficiency reduces monocyte infiltration and plaque lesion area	(3)
Cardiovascular disease	Lack of the P2Y1 receptor decreases leukocyte infiltration into atherosclerotic plaques	(4)
Cardiovascular disease	Neointima injury results in upregulation of the P2Y2 receptor in rats, which in turn promotes leukocyte adhesion	(6, 7)
GvHD	ATP released from damaged cells aggravates GvHD by activation of antigen-presenting cells	(23, 73)
GvHD	P2Y2 deficiency in monocytes reduces GvHD severity by abrogating ERK activation and ROS production	(50)
GvHD	CD73 deficiency increases T cell allo-reactivity and aggravates murine GvHD	(65, 66)
Inflammatory bowel disease	CD39 deletion aggravates chemically induced colitis in mice	(57, 60)
	CD39 expression on Tregs is associated with better therapy response in inflammatory bowel disease patients	
Inflammatory bowel disease	Lack of CD73 aggravates experimental inflammatory bowel disease in mice	(64)
Ischemia–reperfusion injury	CD39 plays a protective role by reducing vascular leakage	(56)
Lupus-associated nephritis	Inhibition of the P2X7 receptor reduces nephritis severity	(17)
Multiple sclerosis	ATP increases oligodendrocyte excitotoxicity and plaque formation via binding the P2X7 receptor	(15, 16)
	Gain of function polymorphisms of the P2X7 receptor are associated with increased MS risk	
Platelet aggregation	Inhibition of P2Y12 signaling blocks platelet aggregation	(10)

## Purinergic Signalling in Airway Inflammation

In the context of airway inflammation, purinergic signaling also plays a significant role in the activation of immune cells (Table 1). For instance, increased ATP levels following allergen challenge recruit airway-specific myeloid cells and induce Th2 cell polarization and eosinophilic airway inflammation, which are major features of allergic asthma in humans. Neutralization of ATP signaling abrogated airway inflammation in response to allergens (11). Further studies emphasized the role of P2X7 receptor expression on dendritic cells (DCs) in this context (12). However, purinergic receptor expression is not limited only to the immune cell compartment. The P2Y6 receptor was found on airway epithelial cells with abundant expression upon allergen challenge and its inhibition by synthetic antagonists or the genetic deletion reduced IL-6 and IL-8 secretion by epithelial cells and improved disease outcome in a murine model (13). More recent studies suggest that purinergic signaling might be regulated by microRNAs. There is evidence that the immunomodulatory miR-155 is necessary for intact purinergic signaling. Lack of miR-155 resulted in impaired DC chemotaxis toward pro-inflammatory stimuli with subsequently reduced airway inflammation in mice (14).

## Purinergic Signaling in Autoimmunity

Purine nucleotide-mediated signaling has been implied in autoimmune diseases, including multiple sclerosis (MS), psoriasis, and nephritis among others (Table 1).

P2X7 receptor activation by ATP binding triggers oligodendrocyte excitotoxicity and increases MS plaque formation in an experimental autoimmune encephalomyelitis model. More importantly, P2X7 receptor upregulation is observed in healthy tissue of MS patients, suggesting that inhibition of purinergic signaling might be a novel therapeutic target (15). In line with these data, a signal nucleotide polymorphism in the human *p2x7r* gene that leads to a gain of function amino acid exchange, occurs more frequently in MS patients than in healthy controls (16). P2X7 receptor upregulation was also observed in lesional and non-lesional skin of psoriasis patients. In lupus-associated nephritis, P2X7 receptor antagonists reduced nephritis severity, pro-inflammatory serum cytokines, and NLRP3 inflammasome activation underlying once more the broad therapeutic potential of these pathways (17).

## The Role of P2X7 in GvHD

**Figure 1:**
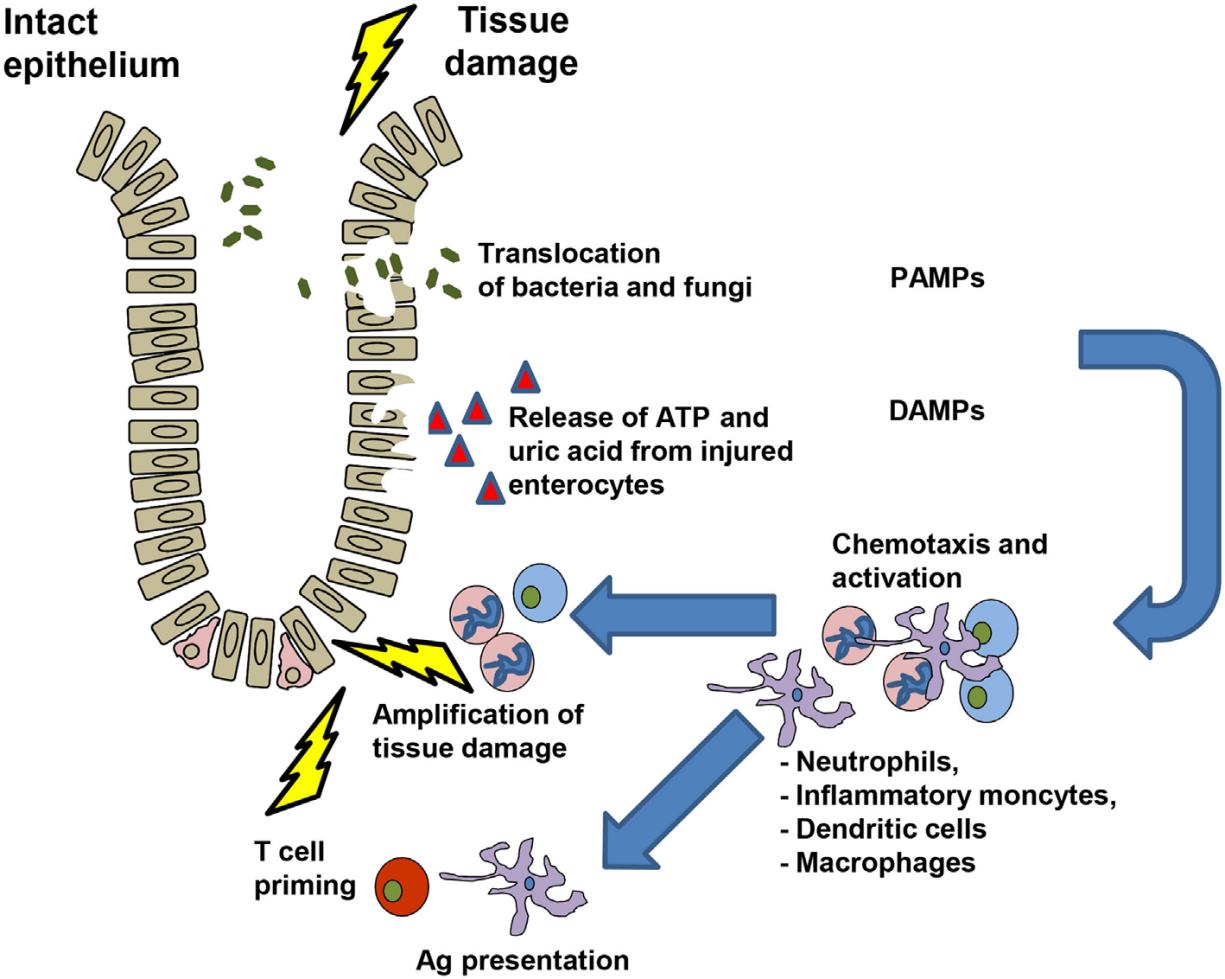
**DAMPs, PAMPs and immune cell interactions during GvHD development**.

ATP is a molecule with a high intracellular concentration that is released upon cell stress. In the absence of tissue damage, the intracellular ATP concentration ranges from 3 to 10 mM, while extracellular ATP levels are as low as 10 nM. This balance is regulated by ectonucleotidases, such as CD39 and CD73, which dephosphorylate ATP to ADP, AMP, and ultimately generate adenosine (2, 18, 19). The P2X7 receptor is a cation channel activated by high concentrations of ATP (20). P2X7 plays a central role for IL-1β secretion via activation of the NACHT, LRR, and PYD domains-containing protein 3 (Nlrp3) inflammasome (21, 22). We observed that release of ATP from damaged cells after allo-HCT amplified acute GvHD (23) via enhanced maturation of APCs and reduced Treg numbers. Besides activation via P2X7, the Nlrp3 inflammasome can be activated by uric acid (24) and Syk signaling (25). We found that uric acid enhanced GvHD in the early phase after allo-HCT (26). Inhibition of Syk reduced GvHD-related mortality in the mouse model without impairing anti-MCV or anti-leukemia responses (27). After tissue damage due to chemotherapy of irradiation, neutrophil granulocytes (neutrophils) and inflammatory monocytes reach a site of inflammation particularly early and participate in the first line of defense. Different studies have shown the chemotactic role of ATP for neutrophil chemotactic recruitment (28, 29). It was shown that purinergic signaling causes strong activation of human neutrophils (30) and activated neutrophils can release ATP through pannexin-1 hemichannels by an active process, which means that the process consumes energy (30). Neutrophils release reactive oxygen species (ROS) upon activation with bactericidal activity and the potential to cause local tissue damage (31, 32) that was shown to enhance GvHD (33) (Table 1). The mechanism as to how DAMPs, PAMPs, and neutrophils may contribute to GvHD is depicted in Figure 1. Besides neutrophils (33–35), other myeloid cell populations in particular DCs (36), macrophages (37), and certain monocyte subsets (38, 39) were found to enhance or reduce GvHD. Different purinergic receptors were found to be expressed by these myeloid cells (2) and their activation modifies the immune response elicited by the respective myeloid cell type. Myeloid-derived suppressor cells (MDSC) that lack a function Nlrp3 inflammasome are more protective against GvHD compared to WT MDSC (40), indicating that a functional Nlrp3 inflammasome modifies the inflammatory phenotype of this myeloid cell type. Besides MDSCs, DCs were shown to be influenced by different signals from purinergic receptors. To present the antigen that was taken up at the site of inflammation by a DC, costimulation is required. ATP is involved in this process as it enhances the maturation of human monocyte-derived DCs with increased levels of costimulatory molecules (41, 42). Recently, the central role of donor-derived colonic CD103^+^ DCs in Ag presentation to donor T cells that then induce GvHD was reported (43). These reports from different groups support the concept that P2X7 activation in myeloid cells enhances their inflammatory phenotype that then promotes T cell priming and inflammation that ultimately lead to GvHD.

## The Role of P2Y2 in GvHD and Inflammation

The activation of P2Y2 was shown to promote tissue damage in airway inflammation (44, 45) and acute liver injury (46). However, P2Y2 was also shown to have protective effects in a model of lung infection induced by pneumonia virus of mice (47). P2Y2 can be activated by different nucleotides, the P2Y2 ligand ATP was found in different inflammatory diseases, including inflammatory bowel disease (48), glomerulonephritis (49), asthma (11), and diabetes (48). We recently reported that *P2y2* deficiency of the recipient caused lower levels of myeloperoxidase in the intestinal tract of mice developing GvHD (50). Selective deficiency of P2Y2 in inflammatory monocytes lead to reduced GvHD severity (50) and *P2y2*^−/−^ inflammatory monocytes had defective ERK activation and ROS production. Besides these results in the mouse model, histochemical analysis of patient samples revealed that the frequency of P2Y2^+^ cells in inflamed GvHD lesions correlated with histopathological GvHD severity.

## Purinergic Signaling and the Graft-Versus-Tumor Effect

Transplanation of the donor immune system into the allo-HCT recipient provides the graft-versus-tumor (GvT) effect that ensures long-term control of the underlying malignancy. Due to the fact that most treatments reduce the activation of the immune system, preserving the GvT effect is a major issue in post-transplant care. Interestingly, reduction of GvHD by application of the broad-spectrum P2R inhibitor PPADS did not interfere with the GvT effect, likely due to the fact that CD8^+^ T cell function is independent of purinergic signaling (23). On the other hand, A2A-AR expression on T cells allows adenosine to reduce allo-reactivity in a murine allo-HCT model, so that blockade of adenosine production allows for a more potent GvT effect at the cost of aggravated GvHD (51). These data suggest that purinergic receptor expression on T cells is crucial for the modulation of the GvT effect and offers a few therapeutic perspectives. On the one hand, in cases where a functional GvT effect is required, such as high-risk malignancies, a P2R inhibition might be a successful approach that reduces GvHD but leaves the GvT effect untouched. On the contrary, when performing allo-HCT in a patient with a benign hematopoietic disease without a necessity for a GvT activity, adenosine signaling might be enhanced as a therapy strategy for GvHD.

## Mechanisms to Counterbalance the Effects of Nucleotides

### Impact of the Ectonucleotidases CD39 and CD73 on Purinergic Signaling

Purine nucleotides are naturally metabolized by ectonucleotidases – cell surface enzymes with catabolic activity in the extracellular space. Two ectonucleotidases have been mainly proposed in the context of inflammation and GvHD so far – CD39 and CD73 (ecto-5′-nucleotidase). While CD39 catabolizes the first two steps in purine metabolization, mainly the dephosphorylation of ATP to ADP and AMP, CD73 is involved in the last step, namely the generation of adenosine from AMP. Adenosine itself is a potent anti-inflammatory mediator that binds to the four receptors belonging to the P1 receptor family and counteracts the effects of the pro-inflammatory ATP (51). Ectonucleotidase activity counterbalances the effects of nucleotides by regulating their concentration in the extracellular space. Concomitant expression of CD39 and CD73 is observed, for example, on regulatory T cells (52) and multipotent mesenchymal stromal cells (53). Activity of soluble recombinant CD39 removes ATP and ADP from the extracellular space and inhibits platelet aggregation *in vitro* (54). However, CD39 seems to play a dual role in hemostasis, as CD39-deficient mice exhibited prolonged bleeding times resulting from P2Y1 receptor desensitization (55). CD39 activity protects in the context of ischemia–reperfusion injury by modulation of vascular leakage (56). Furthermore, CD39 deletion rendered mice more susceptible to chemically induced murine colitis (57).

With regard to GvHD, recent studies demonstrate that CD39 activity on regulatory T cells induces the expression of the A2A-adenosine receptor on conventional T cells (58). Moreover, CD39-mediated adenosine signaling is important for the regulatory T cell-mediated inhibition of NOTCH1 signaling in conventional T cells (58), which is a known protective mechanism in the context of acute GvHD (59). Additionally, higher CD39 levels were found on regulatory T cells of inflammatory bowel disease patients in clinical remission when compared to non-responders (60).

Generation of adenosine from AMP via CD73 is mostly known as an anti-inflammatory reaction that dampens the pro-inflammatory cascades following ATP accumulation. In rheumatoid arthritis, lack of CD73 enhanced disease development, including Th1 cell differentiation, cytokine production, and joint destruction, and this was reversed by administration of a selective A2A-adenosine receptor agonist (61). In addition, decreased levels of CD73 were found on the surface of synovial fluid mononuclear cells in children with juvenile idiopathic arthritis (62). The immunosuppressive role of CD73 is also shown by the fact that mice lacking this molecule are more prone to autoimmune glomerulonephritis (63) and inflammatory bowel disease (64).

In the context of allo-HCT, we and others have previously shown that CD73 and adenosine modulate the severity of GvHD but might also represent a target for the enhancement of the graft-versus-leukemia (GvL) effect (65, 66). In absence of CD73 and adenosine, alloreactive T cells show a stronger proliferation with increased secretion of pro-inflammatory cytokines and improved migration capacity. This more aggressive T cell phenotype translates into more pronounced GvHD severity, but also offers a target for enhancing the GvL effect in the context of allo-HCT (67).

CD73 and adenosine seem to play a differential role in inflammation, depending on the disease model, since recent studies suggest that CD73 might potentiate inflammation in the context of atherosclerotic plaque formation (68) and radiation-induced lung fibrosis (69).

### P1 and P2 Receptor Modulation

The broad role of purinergic signaling in inflammation suggests a great therapeutic potential for compounds which modulate purinergic receptor signaling. P2Y12 receptor blockers, such as clopidogrel have now long been employed for inhibition of platelet aggregation.

Another receptor with a promising role in immune responses is the P2X7 receptor. Preclinical studies have suggested a beneficial role for P2X7 blockade in allograft vasculopathy (70) ischemia–reperfusion injury (71), acute lung injury (72), and GvHD (23, 73) among others. Also blocking downstream effects of P2X7, namely Nlrp3 inflammasome activation reduced GvHD in different models (26, 74, 75). However, first clinical trials with P2X7 receptor antagonists proved to be disillusioning. Phase II clinical trials with the compound AZD9056 could not show a reliable benefit in the treatment of patients with rheumatoid arthritis (76, 77) or Crohn’s disease (78). These data indicate that inhibition of P2X7 receptor signaling might be a powerful target to modulate inflammation but there is still need for development of active compounds for the clinical setting.

Adenosine is the counterpart of the pro-inflammatory nucleotides ATP, ADP, UTP, and UDP. Adenosine is mostly generated among others by regulatory T cells and binds to the four receptors belonging to the P1 receptor family, A1, A2A, A2B, and A3 adenosine receptor. In the context of inflammation, the A2A receptor has been implied as anti-inflammatory in a wide spectrum of preclinical disease models.

A2A receptor agonists showed beneficial effects also in preclinical models of rheumatoid arthritis (79), encephalomyelitis (80), and allergic asthma (81). A2A receptor involvement in GvHD has also been shown by our group and others. A2A receptor expression on alloreactive T cells is critical for the integration of the protective CD73-mediated adenosine signaling (65). Treatment with a selective A2A receptor agonist, ATL146e inhibited T cell activation and reduced GvHD severity (82). These data were confirmed using other A2A receptor agonists with increased frequency of regulatory T cells in the GvHD target tissues (83). Numerous early clinical trials with adenosine receptor agonists are ongoing or have been completed recently, including indications such as psoriasis, rheumatoid arthritis, sickle cell anemia, myocardial reperfusion, and nerve injury (84) and hold promise to become part of the therapeutic arsenal against inflammatory diseases. To interfere with a broad activation signal as it is exerted by nucleotides the inhibition of the central signal is most promising and modification of TCR signaling can lead to Treg development (85). Besides purinergic receptor inhibition promising targets are the γc receptor (86) or Janus kinases (87, 88).

## Summary

Purinergic signaling belongs as a DAMP to the intrinsic mechanisms for inflammation regulation without pathogen exposure. Differential receptor expression is observed on various cell and tissue types, indicating distinct roles of purines depending on the particular disease context. In general, nucleotides as ADP, ATP, UDP, and UTP serve as “alarmins” and activate neutrophil granulocytes, macrophages, DCs, and platelets. On the other hand, adenosine produced by regulatory T cells or mesenchymal stem cells counteracts the effects of the nucleotides by binding to P1 receptors. The findings from multiple groups in different models of pathogenic inflammation indicate a central function of different purinergic receptors, such as P2X7 and P2Y2, in ATP-activated recipient myeloid cells during GvHD, which could be exploited when targeting danger signals to prevent GvHD. Current efforts are concentrating on the development of bioavailable and efficient compounds for the conduct of clinical trials.

## Author Contributions

The review article was designed and written by RZ together with the first author PA. Both performed literature review and critical discussion of the published literature.

## Conflict of Interest Statement

The authors declare that the research was conducted in the absence of any commercial or financial relationships that could be construed as a potential conflict of interest.
